# The Anatomy, Etc. of the Placenta

**Published:** 1860-11

**Authors:** 


ART. IX.—*The Anatomy and Physiology of the Placenta. The Connection of the Nervous Centres of Animal and Organic Life.* By JOHN O'REILLY, M.D., etc. etc. 8vo., pp. 111. New York, 1860.

THE chapters comprised in this brochure were originally published in the *American Medical Gazette*, from which they have been transferred, without any alteration, to their present place. The first paper treats of the anatomy and physiology of the placenta; the second, of the connection of the nervous centres of animal and organic life; and the third, of syphilitic iritis. We regret that want of space prevents us from giving a more full account of Dr. O'Reilly's labors.

